# Secondary Piggyback Intraocular Lens for Management of Residual Ametropia after Cataract Surgery

**DOI:** 10.18502/jovr.v16i1.8244

**Published:** 2021-01-20

**Authors:** Zahra Karjou, Mohammad-Reza Jafarinasab, Mohammad-Hassan Seifi, Kiana Hassanpour, Bahareh Kheiri

**Affiliations:** ^1^Ophthalmic Research Center, Research Institute for Ophthalmology and Vision Science, Shahid Beheshti University of Medical Sciences, Tehran, Iran

**Keywords:** Residual Ametropia, Intraocular Lens Implantation, Piggyback IOL Implantation

## Abstract

**Purpose:**

To investigate the indications, clinical outcomes, and complications of secondary piggyback intraocular lens (IOL) implantation for correcting residual refractive error after cataract surgery.

**Methods:**

In this prospective interventional case series, patients who had residual refractive error after cataract surgery and were candidates for secondary piggyback IOL implantation between June 2015 and September 2018 were included. All eyes underwent secondary IOL implantation with the piggyback technique in the ciliary sulcus. The types of IOLs included Sulcoflex and three-piece foldable acrylic lenses. Patients were followed-up for at least one year.

**Results:**

Eleven patients were included. Seven patients had hyperopic ametropia, and four patients had residual myopia after cataract surgery. The preoperative mean of absolute residual refractive error was 7.20 ± 7.92, which reached 0.42 ± 1.26 postoperatively (*P* < 0.001). The postoperative spherical equivalent was within ±1 diopter of target refraction in all patients. The average preoperative uncorrected distance visual acuity was 1.13 ± 0.35 LogMAR, which significantly improved to 0.41 ± 0.24 LogMAR postoperatively (*P* = 0.008). There were no intra- or postoperative complications during the 22.4 ± 9.5 months of follow-up.

**Conclusion:**

Secondary piggyback IOL implantation is an effective and safe technique for the correction of residual ametropia following cataract surgery. Three-piece IOLs can be safely placed as secondary piggyback IOLs in situations where specifically designed IOLs are not available.

##  INTRODUCTION

Cataract surgery currently plays a pivotal role in achieving the best possible postoperative refraction, resulting in patients' independence from spectacles. Despite the advances in surgical techniques and intraocular lens (IOL) power calculation, residual refractive error and refractive surprise occasionally occur and cause both patients' and surgeons' dissatisfaction.^[1]^

Inaccurate estimation of postoperative IOL position, incorrect biometry measurements, and error in IOL power selection are among the main causes of residual refractive error. Additionally, patients with high ametropia are more prone to residual refractive error, mainly due to the limitations of IOL calculation formula and imprecision of IOL manufacturing in these extreme conditions.^[2]^

There are multiple surgical techniques used to correct residual refractive error. Various factors affect proper method selection, including the amount of residual refraction and experience of the surgeon, while laser refractive surgeries are considered for lower amounts of residual errors. IOL exchange or piggyback lens implantation is required to correct higher amounts of residual errors.^[3,4]^

Piggyback IOL implantation was first introduced in 1993 by Gayton and Sanders^[5]^ and involves the placement of another IOL in the bag or more recently, in the sulcus.^[6]^ Higher safety profile, easier technique, and the potential for removing the second lens are the advantages of piggyback IOL implantation over IOL exchange.^[4,7]^ However, the increased risk of glaucoma, iris pigment release, and intralenticular opacification make this procedure controversial for many surgeons.^[8,9,10,11]^

Piggyback IOL implantation is considered primary when the refractive error is higher than can be corrected with one IOL and secondary when the residual refractive error is corrected. In secondary piggyback IOL implantation, in which the second IOL is placed in the ciliary sulcus, different IOL designs and types are used, including monofocal, multifocal, and toric.^[2]^

The present study aimed to investigate the clinical outcomes and complications of secondary piggyback IOL implantation in a tertiary referral eye center.

##  METHODS

In this prospective interventional case series, all patients who underwent secondary piggyback IOL implantation at Labbafinejad Medical Center from June 2015 to September 2018 were included. The study protocol was approved by the Ethics Committee of the Ophthalmic Research Center, which is the equivalent of the Institutional Review Board at Shahid Beheshti University of Medical Sciences, and adheres to the tenets of the Declaration of Helsinki. Patients who had hyperopic or myopic residual refractive errors following uneventful cataract surgery and had no compliance with spectacle correction were included in the study. The amount of refractive error required for surgery was individualized for each patient and did not have an exact cut-off. Patients with ocular inflammation, iritis, glaucoma, significant guttate or corneal edema, and any complications in the previous surgery that precludes well-centered IOL in the bag were excluded from the study. Informed consent was obtained from all participants.

### Preoperative Assessment and Piggyback IOL Power Calculation 

An experienced optometrist measured the patients' uncorrected distance visual acuity (UDVA) and best-corrected distance visual acuity (BCVA) using the Snellen chart. Subjective refraction was measured and recorded for all patients. In patients with hyperopic residual refractive error, the power of the piggyback IOL was calculated by multiplying the desired spherical equivalent by 1.5. In myopic patients, the power of the IOL was similar to the desired spherical equivalent. This method was described by Gayton et al.^[12]^

The type of IOL selection was individualized for each patient based on their refractive error, IOL availability, and surgeon's experience (Table 1).

### Surgical Technique 

The minimum required interval between the first surgery and piggyback IOL implantation was three months. All procedures were performed by one experienced cornea surgeon (M.J.). Young and uncooperative patients underwent general anesthesia. In other patients, topical tetracaine 0.5% (Anestocaine, Sinadarou, Tehran, Iran) was instilled and coupled with intracameral lidocaine 2%. Using a 2.8-mm keratome, a clear corneal incision was made on the steep meridian. After the formation of the anterior chamber and area behind the iris with the use of viscoelastic, the IOL was inserted into the ciliary sulcus. OVD (Ophthalmic Viscosurgical Devices) was thoroughly washed using an irrigation and aspiration probe. The incision was made watertight using stromal hydration or a nylon 10-0 suture. Subconjunctival antibiotics were injected at the end of surgery.

On postoperative day 1, topical 0.5% chloramphenicol (ChlobioticⓇ, Sina Darou, Tehran, Iran) was started four times a day, and 0.1% betamethasone (Betasonate, Sinadarou, Tehran, Iran) was applied eight times a day. Antibiotics were continued for one week, and betamethasone was tapered off for six weeks based on the postoperative degree of inflammation. The patients were closely monitored in terms of wound leakage, intraocular pressure (IOP), and inflammation.

### Postoperative Assessment

The patients were followed-up on days 1, 3, 7, and 21, and after three and six months postoperatively, and then yearly. Complete ophthalmic examinations, including UDVA, BCVA, slit-lamp biomicroscopy, and funduscopy were repeated at each visit. Any complication was recorded during the patients' follow-up.

### Statistical Analysis 

Frequency (%), mean ± SD, median, and range were used to describe the data. To evaluate the difference between the two sets (before and after the surgery for spherical equivalent and UDVA), paired *t*-test was used. All statistical analyses were performed using SPSS (IBM Corp. Released 2017; IBM SPSS Statistics for Windows, Version 25.0. Armonk, NY: IBM Corp.).

##  RESULTS

Eleven eyes of 11 patients were enrolled in the present study. The mean age of the patients was 39.27 ± 29.28 (range, 0.5 to 71) years, and 72.7% of the patients were male (Table 2). The absolute mean deviation from emmetropia in the entire cohort was 7.20 ± 7.92 diopters (D), with a median of 4.25 (–9.50 to +14.00 D). In seven patients who had hyperopic ametropia, the mean SE before surgery was 6.85 ± 4.06 (+2 to +14) D. In myopic patients, the mean SE was –7.81 ± 2.01 (–9.50 to –5.00) D.

**Table 1:** Comparison of two types of intraocular lenses


**Variable**	**Acrysof**	**Sulcoflex**
**Generic Name **	MA60AC	Sulcoflex Aspheric
**Country**	Switzerland	United Kingdom
**Company**	Alcon	Rayner Intraocular Lenses
**Pieces**	Three-pieces	One-piece
**Overall diameter**	13 mm	14 mm
**Optic diameter**	6 mm	6.5 mm
**Other properties**	Sharp optic edges	Aspheric, Round edged optic
**Lens material**	Hydrophobic acrylic	Rayacryl hydrophilic acrylic
**Haptic angle **	10°	10°

**Table 2:** Patients demographic


	**Mean ± SD**	**Median (range)**
Age	Years	39.27 ± 29.28	47 (0.5,71)
Sex, *N* (%)	Male	8 (72.7%)	
	Female	3 (27.3%)	
Eye, *N* (%)	OD	5 (45.5%)	
	OS	6 (54.5%)	
Type of ametropia, *N* (%)	Hyperopia	7 (64%)	
	Myopia	4 (36%)	
OD, right eye; OS, left eye; SD, standard deviation; N, number			

**Table 3:** Postoperative clinical outcome of the study participants


	**Mean ± SD**	**Median (range)**	**** ***P*** **-value**
Preoperative ARRE (SE)	Diopter	7.20 ± 7.92	4.25 (–9.5,14)	<0.001
Postoperative ARRE (SE)	Diopter	0 ± 0.97	0.42 (–1,2)	
Preoperative UDVA	logMAR	1.13 ± 0.35	1.31 (0.52,1.48)	0.008
Postoperative UDVA	0.41 ± 0.24	0.3 (0.1,0.7)	
Preoperative BDVA	logMAR	0.41 ± 0.21	0.4 (0.1,0.7)	
Preoperative SE	Hyperopic	6.85 ± 4.06	6.5 (2, 14)	<0.001
Postoperative SE	0.28 ± 0.8	0 (–0.5,2)	
Preoperative SE	Myopic	–7.81 ± 2.01	–8.37 (–9.5,–5)	<0.001
Postoperative SE	0.06 ± 2.43	–0.62 (–2 to 3)	
Preoperative IOP	14.09 ± 2.5	14 (11,17)	0.54
Postoperative IOP	14.27 ± 1.67	15 (11,16)	
Complications	None	
ARRE, absolute residual refractive error; UDVA, uncorrected distance visual acuity; BDVA, best-corrected distance visual acuity; SE, spherical error; IOP, intraocular pressure				

**Table 4:** Indications, clinical outcome, and type of implanted IOL in the study participants


**Patient**	**Age/Sex**	**Possible Causes**	**Pre-op UCVA**	**Pre-op BCVA**	**Post-op UCVA**	**Post-op BCVA**	**Targeted SE**	**Pre-op SE**	**Post- op SE**	**Diff SE (Post-op & Targeted)**	**IOL Type/power**
1	6 Mo M	Incorrect keratometry	–	–	–	+3.00	+14.00	+2.00	+1.00	3-piece/ +21.00
2	4 Y F	Known case of PHPV pseudophakic myopic shift	20/800	3/10	2/10	3/10	0.00	-9.50	0.00	0.00	1-piece/ –10.00
3	41 Y F	Biometric error due to chorioretinal coloboma	20/600	5/10	5/10	5/10	0.00	+10.0	0.00	0.00	3-piece/ +15.50
4	71 Y M	Keratometric error due to corneal nebule	2/10	8/10	8/10	8/10	0.00	+5.00	0.00	0.00	3-piece/ +7.50
5	65 Y F	Keratometric error due to KCN	1/10	7/10	7/10	7/10	0.00	+6.50	–0.50	0.00	3-piece/ +10.0
6	70 Y M	Biometric error due to SO	20/400	2/10	2/10	2/10	0.00	+7.00	+0.50	+ 0.50	3-piece/ +10.00
7	47 Y M	(known case of RP) Acceptable RE	3/10	4/10	5/10	4/10	0.00	+2.00	0.00	0.00	3-piece/ +3.00
8	15 Mo M	Known case of PHPV myopic shift	–	–	–	–	+4.00	–500	+3.50	–0.50	1-piece/ –9.00
9	57 Y M	Wrong IOL power, Human error	20/400	4/10	5/10	4/10	0.00	+3.50	0.00	0.00	3-piece/ +5.00
10	11 Y M	Hx of congenital cataract sx, Myopic shift	20/800	2/10	2/10	2/10	0.00	–9.00	0.00	0.00	Sulcuflex/ –10.00
11	64 Y M	Keratometric error due to PMD	1/10	4/10	4/10	4/10	0.00	–7.75	–1.00	–1.00	Sulcoflex/ –8.00
BCVA, best-corrected visual acuity; UCVA, uncorrected visual acuity; SE, spherical error; PHPV, persistent hyperplastic primary vitreous; RP, retinitis pigmentosa; SO, silicon oil; RE, refractive error; sx, surgery; KCN, keratoconus; PMD, pellucid marginal degeneration; IOL, intraocular lens; op, operative; Diff, difference; M, male; F, female; Hx, history of; Mo, month; Y, year											

### Indications for Surgery and Type of IOL

Seven patients with residual hyperopic ametropia and four patients with residual myopia underwent secondary piggyback IOL implantation.

In general, the inability to achieve accurate keratometric data was the most common cause of residual ametropia. The exact causes of inaccurate keratometric data are summarized in Table 3. Other causes include biometric error secondary to chorioretinal coloboma in one patient, biometric error secondary to silicone oil in another patient, and myopic shift following congenital cataract surgery in three patients (Table 4). Three-piece, 6-mm optic, foldable acrylic IOL (AcrySof MA60AC, Alcon Laboratories, Inc.) was placed in seven patients. The main reasons for choosing a three-piece IOL in these patients did not include the availability and cost. In four patients, Sulcoflex piggyback IOL (Sulcoflex; Rayner Intraocular Lenses Ltd, East Sussex, UK) was placed in the ciliary sulcus. The properties of the two IOLs are summarized in Table 1.

### Refractive Outcome and Complications 

UDVA improved in all participants. The mean duration of follow-up was 22.4 ± 9.5 months. The average preoperative UDVA was 1.13 ± 0.35 LogMAR, which significantly improved to 0.41 ± 0.24 LogMAR postoperatively (*P* = 0.008) (Table 2).

**Figure 1:**
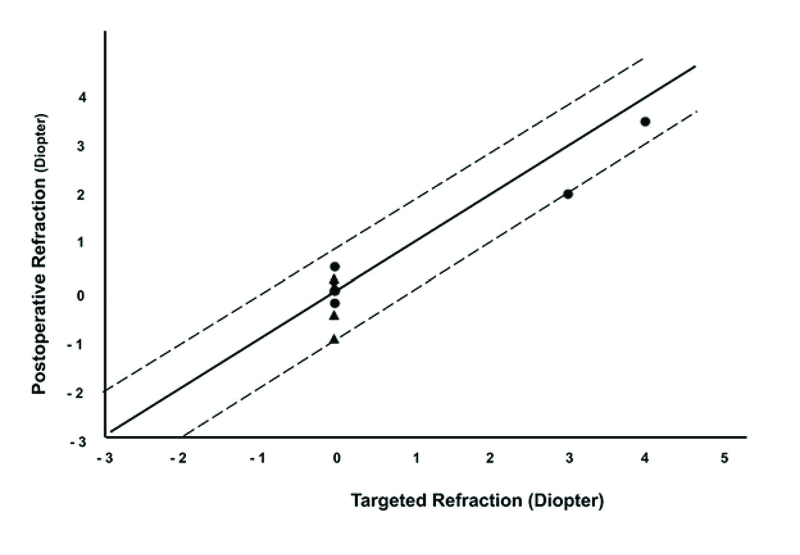
Target refraction plotted against achieved refraction. Triangles depict myopic patients and bullets represent hyperopic patients. All patients were within ± 1 diopters of target refraction.

Postoperative SE was within ± 1 diopter of target refraction in all patients (Figure 1). There was no significant difference between pre- and postoperative IOP (14.09 ± 2.5 mmHg vs 14.27 ± 1.67 mmHg, respectively, *P* = 0.54).

There were no intraoperative complications, including primary IOL and vitreoretinal complications, immediate pupillary block, hyphema, intraocular hemorrhage, and postoperative IOP spike. Similarly, no complications, such as pupillary block, glaucoma, pigment dispersion syndrome, postoperative uveitis, postoperative endophthalmitis, or interlenticular opacification (ILO), were observed during the follow-up period. In follow-up examinations, all IOLs were well centered, and no cases of IOL tilt or capture were observed. In the last follow-up, all patients were satisfied with their quality of vision, and none of them were dependent on spectacles for distance vision.

Patients undergoing either type of IOL had comparable refractive outcomes and complications (Table 4). Postoperative complications such as endophthalmitis and cystoid macular edema did not occur.

### Description of a Presenting Case

A 41-year-old female patient with irido-choroidal coloboma in the left eye was referred to Labbafinejad Medical Center with the complaint of poor vision. She had a history of uneventful cataract surgery and IOL implantation at another eye center three weeks before her presentation to us. Her UDVA was 20/400 in the left eye with the Snellen chart. Her acuity increased to 20/40, with a refraction of +10.50 –1.50 × 150. Slit-lamp biomicroscopy revealed iris and choroidal coloboma in both eyes; it was more severe in the left eye, in which the posterior pole was involved. The cornea was clear. The IOP was within normal limits, and the IOL was well centered in the capsular bag. A review of her previous surgery records revealed implantation of a three-piece acrylic IOL (Acrysof, SA60AT, Alcon, Inc.) with 7.5 diopters calculated based on SRK-T formula. Axial length was measured using a Lenstar LS 900 non-contact biometer (Haag-Streit AG, Switzerland).

An immersion A-scan ultrasound was used to repeat the biometry. A B-scan was also achieved simultaneously. Using the SRK/T formula, the power of IOL was calculated to be 23 diopters, which had a large difference compared with the implanted IOL (15.50 diopters). To calculate the power of the piggyback IOL, the SE was multiplied by 1.5, and the power was calculated to be 15.5 diopters, which was exactly the same as the difference value calculated by biometry.

The patient underwent a piggyback IOL implantation of a three-piece foldable IOL of +15.50 D (MA60AC, AcrySof, Alcon, Inc.) in the ciliary sulcus. Postoperatively, her UDVA reached 20/50. The SE of the residual refractive error was –0.25 D.

##  DISCUSSION

The present study reviewed the indications and clinical outcomes of secondary piggyback IOL implantation at a tertiary referral center over a five-year period. The results revealed that patients with both myopic and hyperopic ametropia following cataract surgery achieved excellent refractive outcomes after the implantation of piggyback IOL in the ciliary sulcus.

Various surgical modalities have been proposed to correct residual ametropia following cataract surgery.^[13]^ Laser refractive procedures, IOL exchange, and secondary IOL implantation are available strategies.^[3]^ Selection of the best one depends on many factors, including the magnitude of residual error and the surgeon's preferences and experience. Laser refractive surgery is an effective and safe method for residual refractive error correction; however, it can create potential complications that may be more common in older patients secondary to concomitant ocular morbidities, such as dry eye and deteriorated wound healing processes.^[14]^ Considering other alternatives, IOL exchange with a new IOL is a very difficult procedure, which requires a high level of expertise, and would impose excessive surgical risk to patients, even if it is performed by an experienced surgeon.^[15]^ Furthermore, this procedure achieves the best results when performed soon before the formation of capsular adhesions, which is not feasible in all patients.^[4,7][10]^

Recently, secondary piggyback IOL implantation has received more attention due to its promising safety profile and easier surgical techniques.^[6,15,16,17,18]^ Additionally, there are many studies reporting predictable refractive outcomes with the application of power calculation of the second IOL, which is not very complicated.^[19]^ Another advantage of a secondary piggyback IOL over IOL exchange is that the implantation of a secondary IOL is a reversible procedure, and if complications such as ILO, pupillary optic capture, pigment dispersion syndrome, or pigmentary glaucoma occur, the removal of piggyback IOL can be considered.^[13]^

The present findings are in line with other studies reporting piggyback IOL implantation. Gayton et al reported an excellent refractive outcome in patients who underwent piggyback IOL implantation.^[16]^ Similarly, they chose a minus-power IOL equal to the patient's residual spherical error. This amount was multiplied by 1.5 in hyperopic patients, regardless of keratometry or axial length.^[12]^ However, there are various methods to calculate secondary IOL power with comparable or even superior results.^[20,21]^

Our patients did not experience any intra- or postoperative complications. Complications of secondary piggyback IOL implantation include ILO, pupillary optic capture, pigment dispersion syndrome, pigmentary glaucoma, and other adverse events that occur generally in ocular surgeries, such as retinal detachment, postoperative endophthalmitis, or uveitis.^[8,9,10,11][22]^ ILO is a unique complication in piggyback implantation, which occurs mainly due to retained regenerative cortical material similar to posterior capsular opacification.^[8,23]^

Recently, the application of different IOL materials and placement of secondary IOL in the ciliary sulcus, which increases the distance between two IOLs, have reduced the incidence of ILO.^[24]^ Accordingly, no ILO was observed in our study series because all secondary IOLs were placed in the ciliary sulcus.

A similar outcome was observed among patients with Sulcoflex IOL compared to patients who underwent three-piece IOL implantation. Secondary piggyback IOLs are available as monofocal, multifocal, toric, and multifocal models.^[1,15,17,18]^ There are three types of IOLs specifically designed for secondary implantation in the ciliary sulcus to correct pseudophakic ametropias or presbyopia: Sulcoflex (Sulcoflex; Rayner Intraocular Lenses Ltd., East Sussex, UK),^[19]^ Add-on (Human optics, add-on IOLs, Germany),^[1]^ and 1st Add-on (1st Gmblt, Mannheim, Germany).^[25]^ In addition, implantable collamer lens and Artiflex phakic IOL are reported to be safely implanted as secondary IOLs.^[18,19,20,26]^ The Sulcoflex, Add-on, and 1st Add-on IOLs were designed to reduce complication rates; no significant difference was observed in our series.^[12]^ These specifically designed IOLs with different powers are not always available, especially in developing countries and countries with a transitional economy. Their cost can also be a concern in these situations. Three-piece IOLs are reported to be safely placed in the ciliary sulcus and capsular bag and are the preferred types of IOL in situations where ciliary sulcus implantation is needed.[< xref  ref - type =" bibr " rid ="B27">27</ xref >],[28] To our knowledge, the use of three-piece IOLs as secondary piggyback implantation has not been previously reported. Herein, we reported their safety and efficacy as secondary piggyback IOL implantation during an approximately two-year follow-up.

Additionally, we described in more detail one of our patients with choroidal coloboma who had refractive surprise after an uneventful cataract surgery. This case highlights the rare possibility of postoperative refractive surprise due to incorrect measurements of the axial length by optical devices, or A-scan without accompanying B-scan, in eyes with posterior pole retinal coloboma or staphyloma.

Although all patients were satisfied with their visual outcomes, the small sample size, lack of matched control group, and relatively short follow-up duration are the important limitations of the current study.

The present study reported the indications and clinical outcomes of a series of patients who underwent secondary piggyback IOL implantation for residual ametropia correction following cataract surgery. This strategy is recommended as an effective and safe technique, especially in extreme ametropia, in the presence of corneal or systemic diseases that exclude laser refractive procedures, or when excimer laser platforms are not available.

##  Financial Support and Sponsorship

Nil.

##  Conflicts of Interest 

The authors have no proprietary or commercial interest in any materials discussed in this article.

## References

[1] Basarir B, Kaya V, Altan C, Karakus S, Pinarci EY, Demirok A. The use of a supplemental sulcus fixated IOL (HumanOptics Add-On IOL) to correct pseudophakic refractive errors. *European journal of ophthalmology* 2012; 22: 898-903. 10.5301/ejo.5000156 22522392

[2] Alio JL, Abdelghany AA and Fernández-Buenaga R. Management of residual refractive error after cataract surgery. *Current opinion in ophthalmology* 2014; 25: 291-297. 10.1097/ICU.0000000000000067 24865171

[3] Jin SX and Lee JK. Refractive surgical corrective options after cataract surgery. *Annals of Eye Science* 2019; 4.

[4] El Awady HE and Ghanem AA. Secondary piggyback implantation versus IOL exchange for symptomatic pseudophakic residual ametropia. *Graefe's Archive for Clinical and Experimental Ophthalmology* 2013; 251: 1861-1866. 10.1007/s00417-013-2283-x 23417295

[5] Gayton JL and Sanders VN. Implanting two posterior chamber intraocular lenses in a case of microphthalmos. *Journal of Cataract & Refractive Surgery* 1993; 19: 776-777. 10.1016/s0886-3350(13)80349-5 8271176

[6] Masket S. Piggyback intraocular lens implantation. *Journal of Cataract & Refractive Surgery* 1998; 24: 569-570. 10.1016/s0886-3350(98)80304-0 9584258

[7] Fernández-Buenaga R, Alió JL, Ardoy AL, Quesada AL, Pinilla-Cortés L, Barraquer RI. Resolving refractive error after cataract surgery: IOL exchange, piggyback lens, or LASIK. *Journal of Refractive Surgery* 2013; 29: 676-683. 10.3928/1081597X-20130826-01 23991761

[8] Gayton JL, Apple DJ, Peng Q, Visessook N, Sanders V, Werner L, et al. Interlenticular opacification: clinicopathological correlation of a complication of posterior chamber piggyback intraocular lenses. *Journal of Cataract & Refractive Surgery* 2000; 26: 330-336. 10.1016/s0886-3350(99)00433-2 10713224

[9] Iwase T and Tanaka N. Elevated intraocular pressure in secondary piggyback intraocular lens implantation. *Journal of Cataract & Refractive Surgery* 2005; 31: 1821-1823. 10.1016/j.jcrs.2005.06.034 16246790

[10] Mehta R and Aref AA. Intraocular Lens Implantation In The Ciliary Sulcus: Challenges And Risks. *Clinical Ophthalmology* 2019; 13: 2317-2323. 10.2147/OPTH.S205148 PMC6885568 31819356

[11] Kim SK, Lanciano Jr RC and Sulewski ME. Pupillary block glaucoma associated with a secondary piggyback intraocular lens. *Journal of Cataract & Refractive Surgery* 2007; 33: 1813-1814. 10.1016/j.jcrs.2007.05.046 17889783

[12] Gayton J and Raanan M. Reducing refractive error in high hyperopes with double implants. Maximizing Results: Strategies in Refractive, Corneal, Cataract and Glaucoma Surgery Thorofare, NJ: SLACK 1996: 139-148.

[13] Sáles CS and Manche EE. Managing residual refractive error after cataract surgery. *Journal of Cataract & Refractive Surgery* 2015; 41: 1289-1299. 10.1016/j.jcrs.2015.05.001 26096522

[14] Battat L, Macri A, Dursun D, Pflugfelder SC. Effects of laser in situ keratomileusis on tear production, clearance, and the ocular surface. *Ophthalmology* 2001; 108: 1230-1235. 10.1016/s0161-6420(01)00623-6 11425680

[15] Trindade FC. Secondary piggyback with PMMA IOL for the correction of refractive surprise after phacoemulsification long-term results of 20 cases. *Revista Brasileira de Oftalmologia* 2013; 72: 8-11.

[16] Gayton JL, Sanders V, Van Der Karr M, Raanan MG. Piggybacking intraocular implants to correct pseudophakic refractive error. *Ophthalmology* 1999; 106: 56-59. 10.1016/S0161-6420(99)90005-2 9917781

[17] McIntyre JS, Werner L, Fuller SR, Kavoussi SC, Hill M, Mamalis N. Assessment of a single-piece hydrophilic acrylic IOL for piggyback sulcus fixation in pseudophakic cadaver eyes. *Journal of Cataract & Refractive Surgery* 2012; 38: 155-162. 10.1016/j.jcrs.2011.06.035 22055074

[18] Sanders DR. Matched population comparison of the Visian Implantable Collamer Lens and standard LASIK for myopia of-3.00 to-7.88 diopters. *Journal of Refractive Surgery* 2007; 23: 537-554. 10.3928/1081-597X-20070601-02 17598571

[19] Falzon K and Stewart OG. Correction of undesirable pseudophakic refractive error with the Sulcoflex intraocular lens. *Journal of Refractive Surgery* 2012; 28: 614-619. 10.3928/1081597X-20120809-01 22947288

[20] Habot-Wilner Z, Sachs D, Cahane M, Alhalel A, Desatnik H, Schwalb E, et al. Refractive results with secondary piggyback implantation to correct pseudophakic refractive errors. *Journal of Cataract & Refractive Surgery* 2005; 31: 2101-2103. 10.1016/j.jcrs.2005.05.023 16412922

[21] Holladay JT, Gills JP, Leidlein J, Cherchio M. Achieving emmetropia in extremely short eyes with two piggyback posterior chamber intraocular lenses. *Ophthalmology* 1996; 103: 1118-1123. 10.1016/s0161-6420(96)30558-7 8684803

[22] Chang SH and Lim G. Secondary pigmentary glaucoma associated with piggyback intraocular lens implantation. *Journal of Cataract & Refractive Surgery* 2004; 30: 2219- 2222. 10.1016/j.jcrs.2004.03.034 15474839

[23] Werner L, Apple DJ, Pandey SK, Solomon KD, Snyder ME, Brint SF, et al. Analysis of elements of interlenticular opacification. *American journal of ophthalmology* 2002; 133: 320-326. 10.1016/s0002-9394(01)01405-2 11860967

[24] Gills JP and Fenzl RE. Piggyback intraocular lens implantation. Developments in ophthalmology 2002; 34: 209-216. 10.1159/000060799 12520616

[25] Reiter N, Werner L, Guan J, Li J, Tsaousis KT, Mamalis N, et al. Assessment of a new hydrophilic acrylic supplementary IOL for sulcus fixation in pseudophakic cadaver eyes. *Eye* 2017; 31: 802. 10.1038/eye.2016.310 PMC5437322 28106890

[26] Akaishi L, Tzelikis PF, Gondim J, Vaz R. Primary piggyback implantation using the Tecnis ZM900 multifocal intraocular lens: case series. *Journal of Cataract & Refractive Surgery* 2007; 33: 2067-2071. 10.1016/j.jcrs.2007.07.032 18053906

[27] Hayashi K and Hayashi H. Comparison of the stability of 1-piece and 3-piece acrylic intraocular lenses in the lens capsule. *Journal of Cataract & Refractive Surgery* 10.1016/j.jcrs.2004.06.042 15767155

